# The Rheolaser Master™ and Kinexus Rotational Rheometer^®^ to Evaluate the Influence of Topical Drug Delivery Systems on Rheological Features of Topical Poloxamer Gel

**DOI:** 10.3390/molecules25081979

**Published:** 2020-04-23

**Authors:** Maria Chiara Cristiano, Francesca Froiio, Antonia Mancuso, Federica De Gaetano, Cinzia Anna Ventura, Massimo Fresta, Donatella Paolino

**Affiliations:** 1 Department of Experimental and Clinical Medicine, School of Pharmacy and Nutraceuticals, University “Magna Græcia” of Catanzaro, Campus Universitario “S. Venuta”-Building of BioSciences, Viale S. Venuta, I-88100 Germaneto-Catanzaro, Italy; mchiara.cristiano@unicz.it (M.C.C.); f.froiio@unicz.it (F.F.); 2 Department of Health Sciences, School of Pharmacy and Nutraceuticals, University “Magna Græcia” of Catanzaro, Campus Universitario “S. Venuta”-Building of BioSciences, Viale S. Venuta, I-88100 Germaneto-Catanzaro, Italy; antonia.mancuso@unicz.it (A.M.); fresta@unicz.it (M.F.); 3 Department of Chemical, Biological, Pharmaceutical and Environmental Sciences, University of Messina, Viale Ferdinando Stagno D’Alcontres 31, I-98166 Messina, Italy; fedegaetano@unime.it (F.D.G.); caventura@unime.it (C.A.V.)

**Keywords:** Poloxamer P407, Rheolaser Master™, Kinexus Rotational Rheometer, skin application, ethosomes, transfersomes, niosomes

## Abstract

Poloxamer 407 copolymer is a versatile and widely used thermo-reversible material. Its use has many advantages, such as bio-adhesion, enhanced solubilization of poorly water-soluble drugs and many applications fields like oral, rectal, topical, nasal drug administration. Hydrogels made up of Poloxamer 407 are characterized by specific rheological features, which are affected by temperature, concentration and presence of other compounds. A strategic approach in topical therapeutic treatments may be the inclusion of drug delivery systems, such as ethosomes, transfersomes and niosomes, into hydrogel poloxamer formulation. The evaluation of the interaction between colloidal carriers and the Poloxamer 407 hydrogel network is essential for a suitable design of an innovative topical dosage form. For this reason, the Rheolaser Master™, based on diffusing wave spectroscopy, and a Kinexus Rotational Rheometer were used to evaluate the influence of nanocarriers on the microrheological features of hydrogels. The advantages of the Rheolaser Master™ analyzer are: (i) its ability to determine viscoelastic parameter, without altering or destroying the sample and at rest (zero shear); (ii) possibility of aging analysis on the same sample. This study provide evidence that vesicular systems do not influence the rheological features of the gel, supporting the possibility to encapsulate an innovative system into a three-dimensional network.

## 1. Introduction

Poloxamer 407 copolymer, also known as Pluronic F127^®^, is a non-toxic and non-irritable polymer which represents an excellent excipient, compatible with many different substances and useful for different purposes, for instance increasing the solubility of poorly water-soluble drugs. Poloxamer 407, due to its thermo-gelling property, is used as a component of pharmaceutical dosage forms for rectal, nasal and oral applications, as well as for cosmetic use. The thermogelling phenomenon is reversible and occurs at a specific sol-gel transition temperature (T_sol →gel_), namely the temperature increase favors the interaction between different segments of the copolymer, thus eliciting the aggregation of Poloxamer 407 molecules into micelles due to the dehydration of the hydrophobic Poloxamer 407 blocks and hence the sample gelation [1].

The T_sol →gel_ is strongly influenced by the Poloxamer 407 concentration [2] and hence the concentration is selected as a function of the use requirements. For example, gels designed for topical use have to be prepared using high Poloxamer 407 concentrations thus obtaining a gel at room temperature. Topical Poloxamer 407 gel is of particular interest since concentrated solutions (>20% *w*/*w*) of the copolymer are transformed from low viscosity transparent solutions to solid gels with a slight heating [3]. However, the gelling temperature and rheological features of gels can be also influenced by other factors, such as presence of drugs, other excipients and other components as drug delivery systems.

The topical use of Poloxamer 407 gels is encouraged because of its non-occlusive behavior at body temperature and its safety [4]. Moreover, its good adhesive property brings some advantage over other gelling agent, increasing the residence time on the skin [5]. The poloxamer 407 gel is well flowing on the skin and has the ability to increase topical permeation of drugs like ketoprofen and piroxicam [6,7]. In principle, the percutaneous adsorption of active ingredients can be further enhanced using Poloxamer 407 hydrogel as a matrix for the dispersion of colloidal drug delivery systems, thus increasing the skin contact time of the colloidal carrier.

Flexible colloidal drug carriers, such as ethosomes, transfersomes and niosomes, have many advantages in comparison with traditional topical dosage forms, thus showing the ability of overcoming the skin barrier and hence enhancing the drug permeability through the stratum corneum [8]. The increased percutaneous drug permeability is due to the deformability of these colloidal carriers, which is provided by the presence in the carrier composition of ethanol, edge activators and liquid lipid components in the case of ethosomes [9], transfersomes [10] and niosomes [11], respectively.

Topical colloidal carriers embedded in the Poloxamer 407 hydrogel can be useful to boost the topical delivery of drugs, owing to the increase of the contact time between the skin and the topical colloidal carriers ensured by the non-occlusive hydrogel as well as the combination of the topical effects of both devices [5]. Normally, the viscosity of the ethosomes, transfersomes and niosomes suspension was found to be low, which was not appropriate for transdermal application. When the colloidal suspension was combined with a gel matrix, it resulted in formation of gelling systems having a high viscosity [12]. Moreover, the hydrogel based on Poloxamer 407 can be easily removed by contact with cool water, thus becoming appreciated by the user [13].

The efficacy of ethosomes, transfersomes and niosomes as topical drug delivery systems is widely described in the literature, but the practical application of these formulations on the skin has low efficiency [14,15]. Therefore, the inclusion of these vesicular systems into three-dimensional network could be useful to guarantee their great residence time on the skin.

In this paper, multi-speckle diffusing wave spectroscopy (MS-DWS) analysis was carried out in association with rheology studies to evaluate the possible changes in rheological features and in gelling temperature of different Poloxamer 407 hydrogels when colloidal carriers were embedded. MS-DWS data, elasticity index and solid-liquid balance determination were investigated using the Rheolaser Master™, which allows no manipulation and no mechanical stress to be applied to the sample [16]; in fact, the Rheolaser Master™ is based on a passive microrheology analysis and the sample can be recovered. This technique allows the monitoring of sample evolution like gelation, rheology ageing and stability. These are parameters which are increasingly important for a number of applications in different fields. Since it is not invasive, this analysis is ideal for the study of soft and frail materials.

## 2. Results and Discussion

### 2.1. Physicochemical and Technological Characterization of Nanosystems

Physicochemical and technological characterization is a fundamental step in the design of drug delivery systems. Mean size, zeta potential and storage stability can influence the final biopharmaceutical feature of dosage forms. In particular, a small size, a narrow size distribution and a negative zeta potential are highly desirable features for colloidal drug carriers to be proposed for topical skin application thus favoring the interaction between nanosystems and skin.

Several possible compositions of all the chosen nanosystems are reported in literature and therefore we decided to prepare ethosomes, transfersomes and niosomes made up of the most representative lipid components [17,18,19]. These nanosystems are the most studied topical drug delivery systems. Scientific literature is rich in works that testify to their efficacy as permeation enhancers for many drugs [20,21,22].

As reported in Table 1, photon correlation spectroscopy showed suitable physico-chemical parameters of the various nanosystems. Namely, sample mean sizes having a narrow range and below 200 nm, independently of the composition. Polydispersity index values were close to 0, thus confirming a narrow size distribution [23]. The negative surface charge of the investigated nanosystems allowed a colloidal particle repulsion, thus avoiding any aggregation phenomenon and hence ensuring a certain colloidal stability [24].

**Figure 1:**
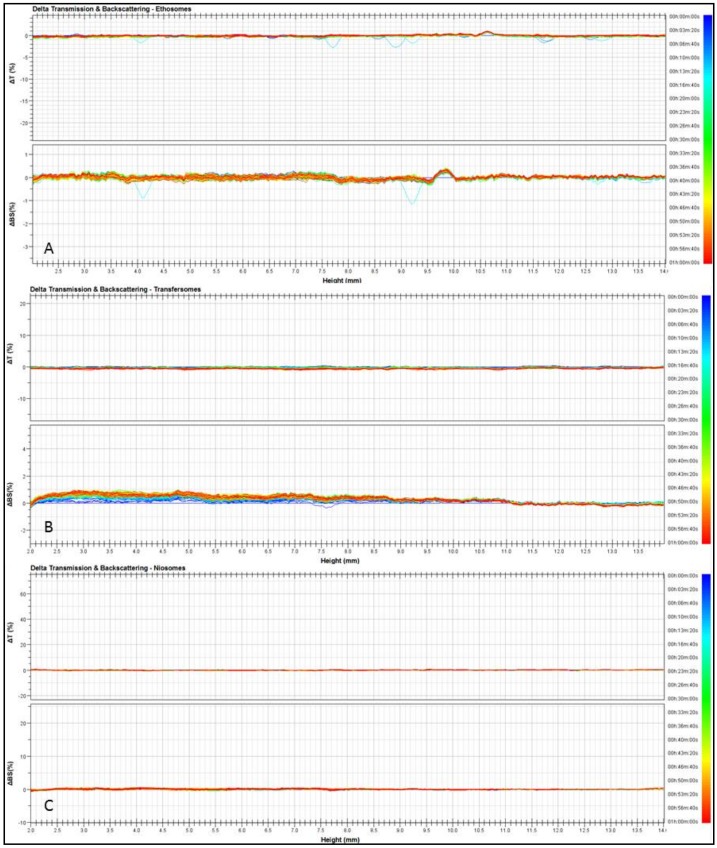
Transmission and backscattering profiles of ethosomes (**A**), transfersomes (**B**) and niosomes (**C**) determined by Turbiscan Lab^®^ Expert. Various runs were representative of three independent experiments. Data are reported as a function of time (0–1 h) and sample height.

**Figure 2:**
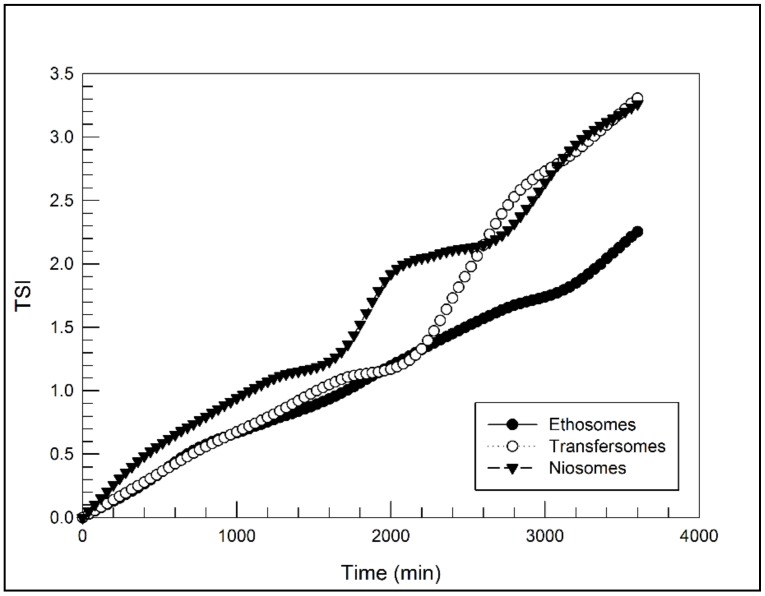
Turbiscan Stability Index (TSI) in function of time obtained for ethosomes, transfersomes and niosomes, by using Turbiscan Lab^®^ Expert.

To confirm the predicted colloidal stability by dynamic light scattering, the three different nanosystems were submitted to Turbiscan Lab^®^ Expert analysis, according to previous procedure [25]. As shown in Figure 1, the transmission and backscattering profiles of ethosomes, transfersomes and niosomes were close to the base line values during experiment (1 h). These findings provide evidence of a homogeneous opalescent aspect of the three nanosystems and the absence of any segregation phenomenon, as further demonstrated by the stability kinetic profiles (Figure 2) which were within a narrow range of TSI (Turbiscan stability index).

### 2.2. Gelation Temperature of Poloxamer Solutions

Gelation temperature (T_gel_) corresponds to the temperature at which the poloxamer solution transforms itself into a hydrogel. According to the proposed topical application, the Poloxamer 407 samples have to be in the hydrogel form at room temperature. Therefore, different amounts of Poloxamer 407 were used for the preparation thus modulating the T_gel_ as reported in the Supplementary Materials, Table S1. The T_gel_ decreased following the increase of the Poloxamer 407 concentration [26]. In particular, solutions containing 15% or 17% of Poloxamer 407 did not form a hydrogel at a suitable temperature for skin topical applications; while, greater concentration of Poloxamer 407 allowed forming hydrogels at room temperature.

This finding could be explained by a configuration change, such as, at low temperature and in dilute solution, poloxamer molecules exist as unimers, but with an increase of solution temperature and polymer concentration a micellization occurs [27]. In the case of more concentrated poloxamer solutions, the poloxamer macromolecules can be self-arranged into a close-packed meander network, thus forming a viscous hydrogel.

Taking into consideration the T_gel_ data, samples prepared with Poloxamer 407 at 20%, 25% and 30% (*w*/*w*) concentrations were selected for further investigations in the presence of various nanosystems and evaluation of their influence on the gelation point was again performed. By these experiments, it was observed that the presence of nanosystems elicited no significant effect on the Poloxamer 407 T_gel_ (data not shown).

### 2.3. Microrheology Characterization by Rheolaser Master

The microrheology of samples was evaluated with the Rheolaser Master™ using diffusing wave spectroscopy. Standard numerical algorithms were used to deduce the statistical parameters of the sample [28]. In micro-rheology, the particles contained in the sample were used to probe the fluid and measure local deformations due to the Brownian movement. The movement of particles causes the deformation of speckle images and the detector records the dynamics of the speckle pattern deformation to quantify the velocity of movement of the particles in the fluid [29].

In a purely viscous hydrogel, the displacement of the particles increases linearly with time, because they have complete freedom of movement within the medium. In this case, the slope of the MSD-vs-decorrelation time curve is directly proportional to the viscosity of the medium. In a visco-elastic hydrogel, the particles are limited in their displacement due to the three-dimensional network and hence the movement of the particles is slowed down, thus decreasing the slope of the MSD curve until a plateau is reached depending on the decorrelation time [30,31]. The decorrelation time can be assimilated to frequency. Namely, the inverse of decorrelation time (1/*t*) corresponds to a specific frequency and, because the particles move according to the rheological features of the hydrogel. A MSD curve represents the viscoelastic behavior of the hydrogel in a frequency domain and it is comparable to a frequency gradient analysis (frequency sweep) in traditional rheology. However, the main difference between the micro-rheological analysis carried out by the Rheolaser Master™ and dynamic rheology is the possibility to analyze at rest the sample, without any modification and stress applied to the sample; therefore, the results obtained are the consequence of a static analysis.

**Figure 3:**
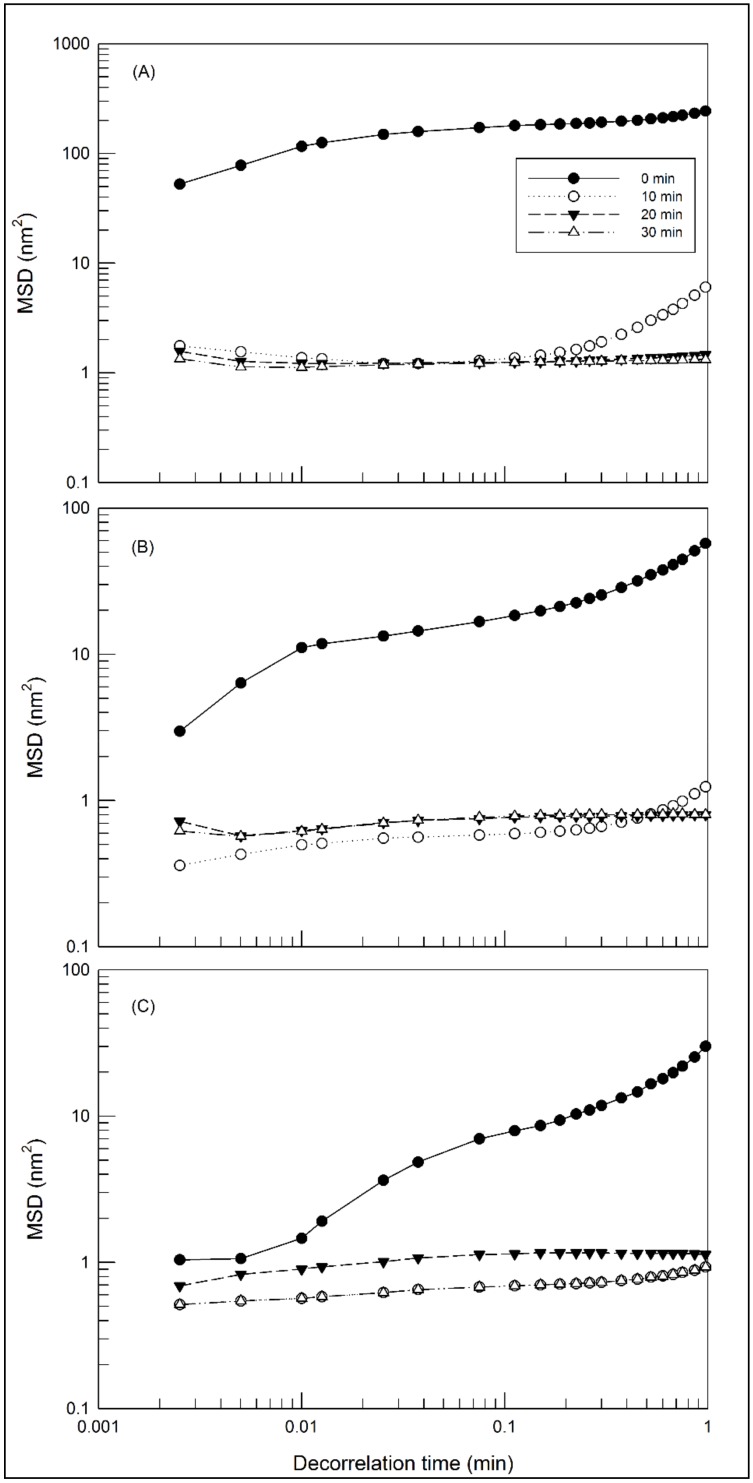
Mean square displacement MSD of hydrogel prepared with different concentrations of poloxamer 407: 20% (*w*/*w*) (**A**), 25% (*w*/*w*) (**B**) and 30% (*w*/*w*) (**C**), as a function of decorrelation time. The illustrated results were representative of three independent experiments.

Figure 3 shows the MSD curves at different aging times of hydrogels prepared with different concentrations of Poloxamer P407. As shown in the figure, the slope of MSD curves decreases as the sample adapts to the analysis temperature. In fact, all samples after 10 min underwent the temperature-dependent gelling process, as evidenced by the reduction of particles’ movement thus eliciting a progressive lowering of the slope of MSD curves. The initial slope MSD values are dependent on the amount of poloxamer. For instance, the slope of MSD curves decreases as the amount of poloxamer increases according to the following decreasing order: Poloxamer 407 20% (*w*/*w*) > Poloxamer 407 25% (*w*/*w*) > Poloxamer 407 30% (*w*/*w*). These results indicate the different freedom of movement of particles in the hydrogels formed starting from the different poloxamer solution. When gelling process is completed, the MSD curves are close to the baseline.

Starting from MSD, considered as the fingerprint of viscoelastic hydrogels [32], other parameters can be obtained thanks to RheoSoft Master^®^ 1.4.0.0 software, the SLB (solid liquid balance) value and the elasticity index (EI) [28]. SLB is directly proportional to the viscoelastic properties of the hydrogel and provides information on the relationship between solid-like and liquid-like behavior of the hydrogel. In fact, SLB allows evaluating the hydrogel adhesiveness, spreadability and gelling point. SLB suitably shows the moment or condition at which gelling occurs. SLB can have values ranging from 0 to 1 and a SLB value of ≅ 0.5 is considered critical for the transformation from a liquid feature to solid feature [33].

**Figure 4:**
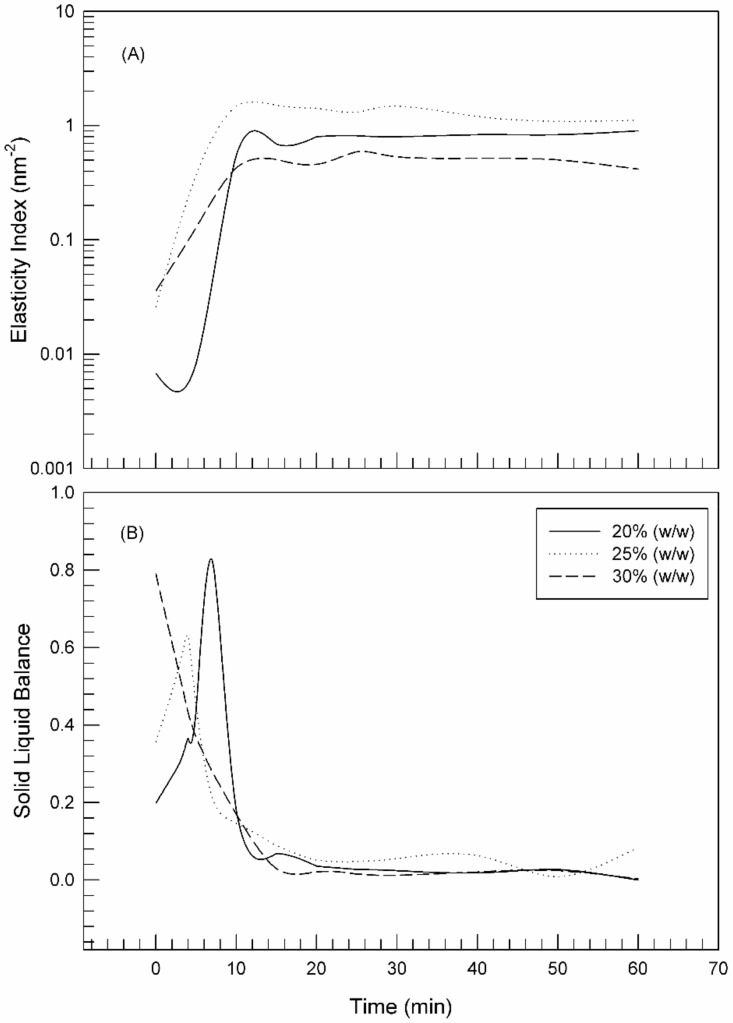
Elasticity Index (EI) (**A**) and Solid Liquid Balance (**B**) profiles versus time for 20% poloxamer 407, 25% poloxamer 407 and 30% poloxamer 407 samples.

When the cold samples were placed at 25 °C, the gelling process started at a rate dependent on the poloxamer concentration, as already shown. In particular, as shown in Figure 4B, the trend of SLB curves showed the evolution from the liquid-like phase to the solid-like one. The SLB profile of 30% poloxamer 407 hydrogel started a decreasing phase with a reduction of SLB values below 0.5 immediately after the sample placement at room temperature (*t* = 13 s); thus, indicating that the sol-gel transition occurred instantaneously. The gelling process occurred at lower rates by reducing the poloxamer concentrations. The reduction of SLB values started at 3 min and 50 s, and 7 min and 20 s in the case of 25% and 20% poloxamer 407 samples, respectively.

The elasticity index (EI) provides information on the hardness, the mesh size and the structure recovery following an effort. The EI value allows easy comparison of many products by evaluating the elastic component, thus classifying quickly and easily the structure. The elasticity index (EI) is the inverse of the MSD plateau value [34]; namely, an increase in the plateau corresponds to a reduction of the network mesh and hence to an increase in elasticity.

As shown in Figure 4 panel A (EI values), the plateau of curves was reached at different rates depending on the poloxamer concentration, but no significant difference was observed for the maximum value, thus indicating that the three poloxamer hydrogels have the same elastic behavior.

The same MS-DWS tests were carried out after the addition of 2% (*w*/*w*) of nanocarriers into poloxamer 407 hydrogels, in order to evaluate whether the micro-rheological characteristics of the hydrogel could be influenced by the presence of the nanocarriers. The topical drug delivery systems were added to the poloxamer solution at 4 °C and left to stabilize overnight. The analyses were carried out in the same conditions used for Poloxamer 407 hydrogels.

**Figure 5:**
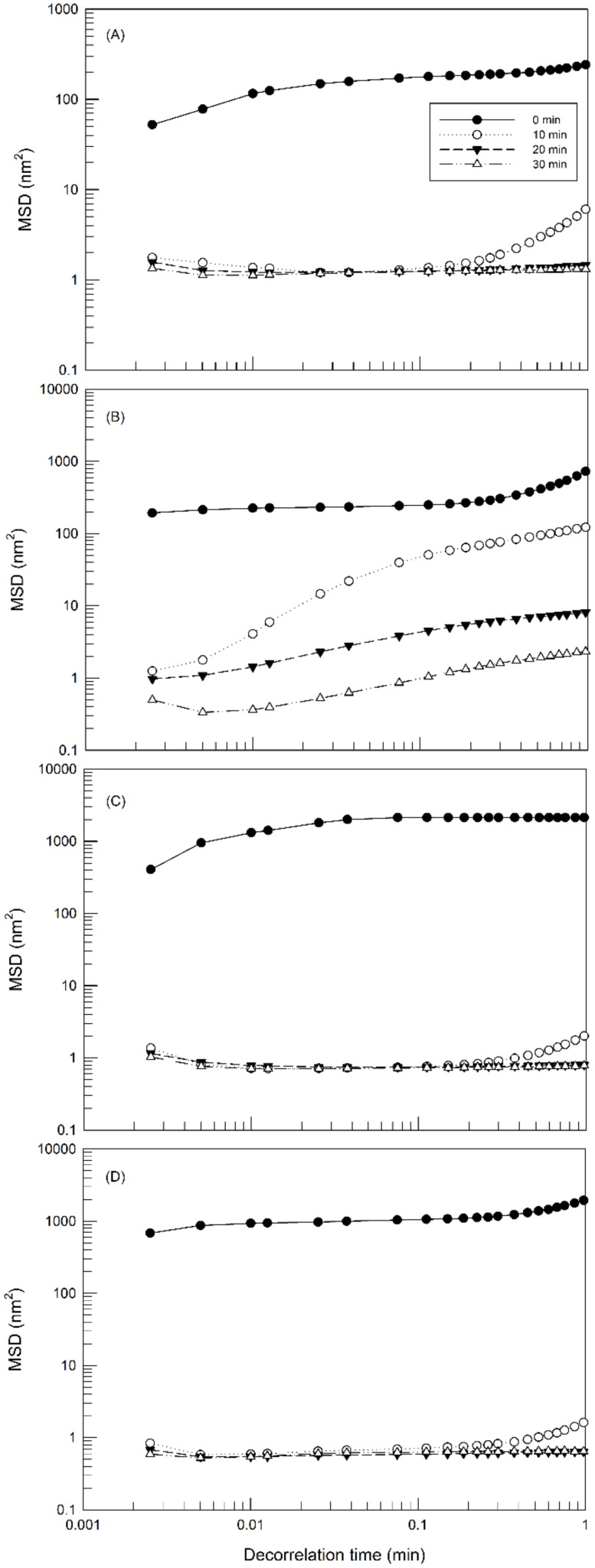
Mean square displacement of hydrogels made up of poloxamer 407 at 20% (*w*/*w*) alone (**A**) or in the presence of ethosomes (**B**), transfersomes (**C**) and niosomes (**D**) as a function of decorrelation time. The illustrated results were representative of three independent experiments.

**Figure 6:**
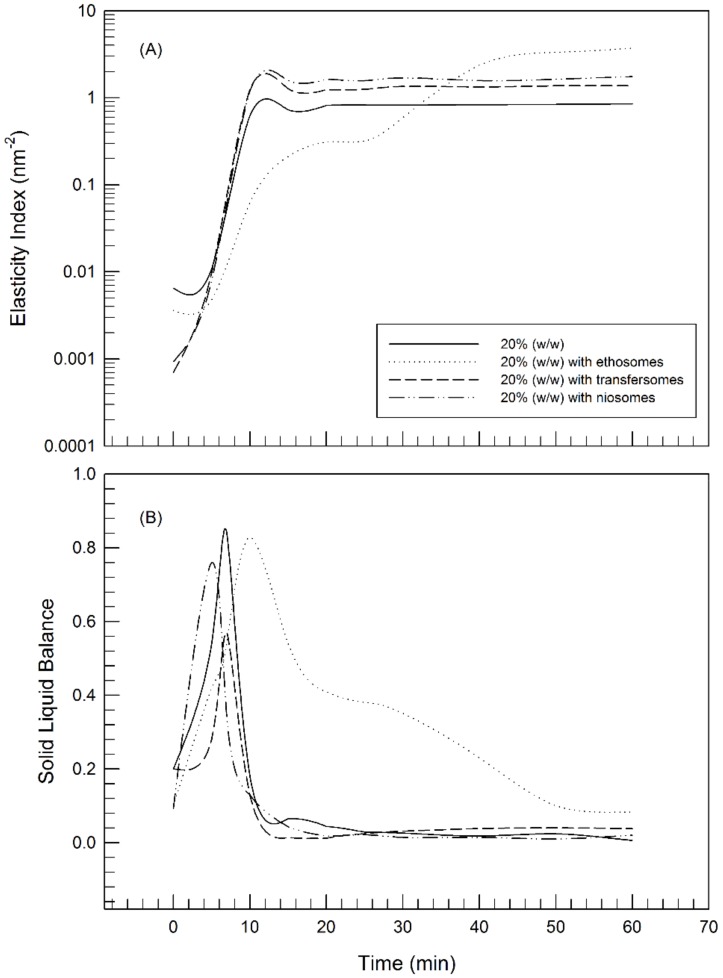
Elasticity Index (EI) (**A**) and Solid Liquid Balance profile (**B**) versus time for 20% poloxamer 407 with and without TDDSs.

As shown in Figure 5, MSD curves confirmed the characteristic sol-gel transition of poloxamer solutions when they are placed at 25 °C. An increase of medium viscosity occurred and, consequently, the particles movements have slowed down. This modify was highlighted by a progressive reduction of the plateau height in MSD curves. The presence of transfersomes (Figure 5C) and niosomes (Figure 5D) poorly influenced the hydrogel network by showing no significant change of the particle movements within the hydrogel of poloxamer 407 at 20% (*w*/*w*), in comparison with MSD curves of unloaded hydrogels. In fact, gels with transfersomes and niosomes seem to take the same time as unloaded samples to gel when placed at 25 °C. On the other hand, the presence of ethosomes (Figure 5B) has slowed the gelation process, hence more time is needed to reach complete gelation of the sample in the presence of ethosomes. The presence of ethosomes within poloxamer gel induced a reduction of the EI values (Figure 6), and hence of the elasticity of the thermo-sensitive hydrogel. Probably, this finding was due to the presence of high ethanol concentration in the composition of the ethosomes, which determined the structuring of a gentler three-dimensional network, than the poloxamer 407 hydrogels. In detail, this aspect may be explained by the presence in ethanol molecule of weak hydrogen bonding and one hydroxyl group that cause weaker bonding in poloxamer gel base [35]. By considering that the presence of ethosomes slowed down the gelling process but did not hamper it, hence this system could be taken into consideration as a more malleable functionalized hydrogel.

Regarding EI and SLB curves, the effects of transfersomes and niosomes on microrheological characteristics of poloxamer gel were less marked and different compared to ethosomes. In particular, EI curves of 20% Poloxamer gel with transfersomes and niosomes reached higher value compared to blank hydrogel, It is possible that these highly flexible and ultra-deformable systems can intervene during the formation of the three-dimensional network, interacting with the polymeric micelles and determining the formation of meshes with smaller dimensions that translate macroscopically with the establishment of more elastic systems.

These effects of nanocarriers on microrheological features of hydrogels were modulated by the amount of poloxamer used for the preparation. In fact, no effect on the microrheological features was observed at 25% (*w*/*w*) and 30% (*w*/*w*) poloxamer 407 concentrations (see Supplementary Materials), probably because the greatest concentrations of poloxamer hid the TDDSs effects.

### 2.4. Dynamic Rheological Characterization of Hydrogels

The microrheological analysis previously described does not provide information regarding the response of the samples to a possible solicitation; to achieve this a dynamic rheological characterization is necessary.

Generally, to measure rheological behavior, a controlled, well-defined deformation or strain is applied to a material over a given time and the resulting force response is measured. In this way it is possible to quantify the functional relationships between deformation, stresses, and the resulting rheological properties such as viscosity, elasticity or viscoelasticity. In our investigation, using the rheometer, two different measures were carried out, (i) viscosity measurements for evaluating the sliding properties, and (ii) oscillatory measurements for evaluating the deformability properties of the hydrogels, without however reaching the sliding. It is possible to apply a specific shear stress or shear rate to investigated hydrogels, to modify the temperature and hence to evaluate the rheological features of hydrogels in terms of viscosity, *G′* and *G″*, and G*. *G′* is the modulus related to the storage of energy during the process or elastic energy; *G″* is the modulus related to the dissipation of energy during the process or viscous energy [36].

The topical skin application of hydrogels requires suitable rheological characteristics [13]. Namely, the hydrogels must have a suitable viscosity thus allowing (i) the formulation to withstand the applied stress during its spreading, (ii) a reversible change which facilitates the consumer for an easy application and guarantees the return to the original viscosity values and (iii) the permanence on the skin when the solicitation ceases. These requirements can be suitably evaluated only after hydrogel characterization in terms of dynamic rheology, which provides information on the viscoelastic behavior of hydrogels prepared both in the absence and in the presence of the investigated TDDSs.

**Figure 7:**
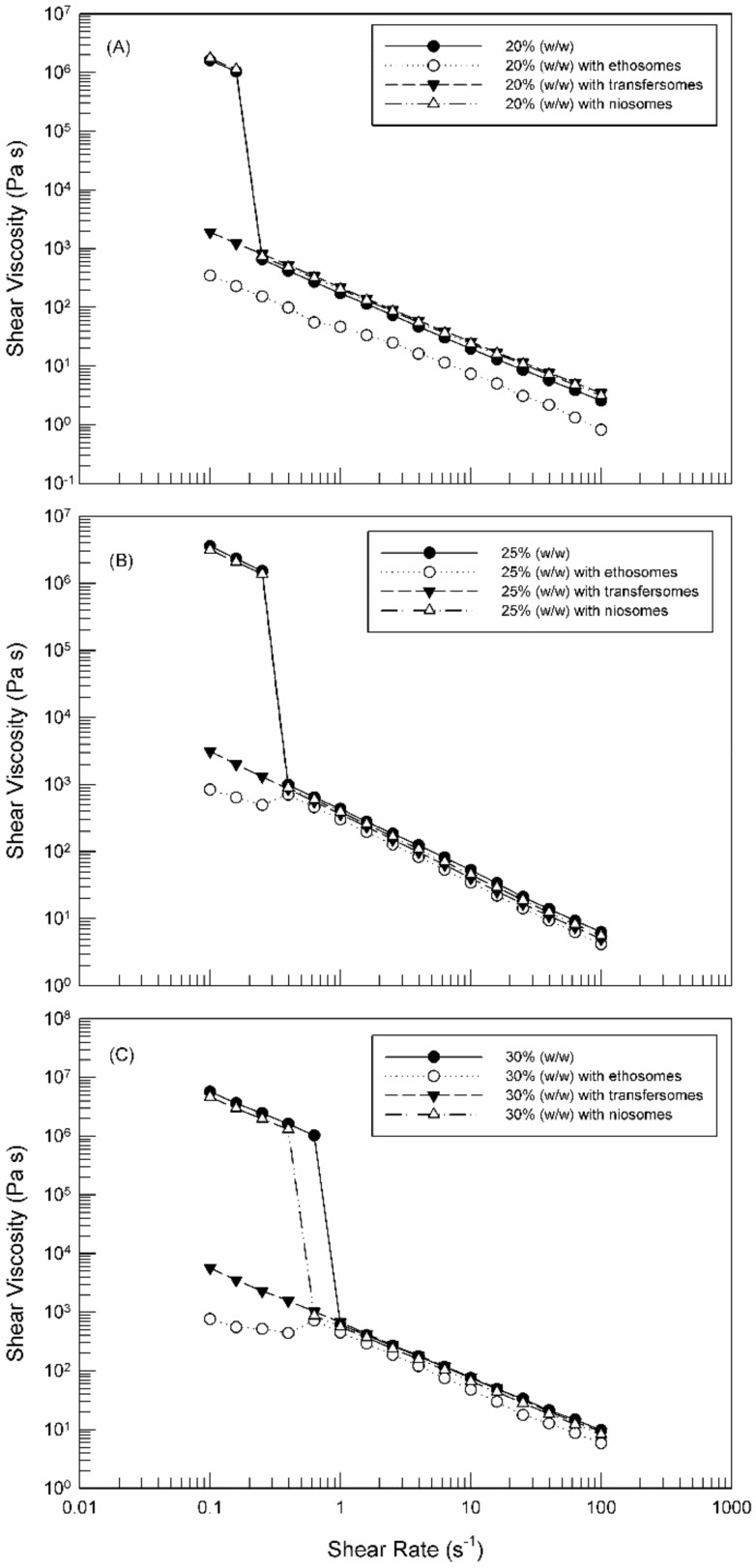
Flow curves (shear viscosity versus shear rate) of (**A**) 20% Poloxamer 407, (**B**) 25% Poloxamer 407 and (**C**) 30% Poloxamer 407, with and without carriers. The illustrated results were representative of three independent experiments.

Figure 7 shows the flow curves of hydrogels prepared with different amount of poloxamer 407, with and without nanosystems, as a function of the applied shear rate. Poloxamer 407 hydrogels were characterized by a thinning shear behavior. Their viscosity decreases with the increase of shear rate [37]. In detail, the viscosity suddenly collapsed when a specific shear rate value (yield point) was reached and exceeding this value the viscosity linearly decreased, regardless of used poloxamer concentration. This trend is important to guarantee a good spread-ability of hydrogel after skin application.

While the rheological profile was independent on the poloxamer 407 concentration, the shear viscosity and yield point value were strongly influenced (Table 2 and Table 3); an increase in the shear rate necessary to collapse the structure corresponds to an increase in the percentage of Poloxamer 407. Accordingly, to our MSD findings, the initial and final viscosity values increased as a function of the poloxamer concentration.

These observations (Figure 7) are in agreement with the microrheological data obtained by the Rheolaser Master. The shear viscosity of hydrogels at 25% (*w*/*w*) and 30% (*w*/*w*) poloxamer 407 concentrations was not affected after the yield point by the presence of TDDSs; in fact, the flow curves of hydrogels were overlapping. Before the yield point, when hydrogels were subjected to low shear rate values, both ethosomes and transfersomes significantly influenced the viscosity of hydrogels (*p*-value < 0.05), thus eliciting a fluidizing effect and hence a reduction of the initial hydrogel viscosity. This effect is probably due to the presence of ethanol in their composition. On the contrary, niosomes elicited no influence on the initial viscosity of the Poloxamer 407 hydrogels at all tested poloxamer concentrations.

According to elasticity index and solid liquid balance findings, ethosomes influenced even more the viscosity of 20% (*w*/*w*) poloxamer hydrogels, which was much lower than the viscosity of the empty hydrogel in the entire tested shear rate interval, thus confirming the formation of a more stress-sensitive hydrogel structure [35].

The oscillation test is another useful rheological assay to characterize a hydrogel. In particular, frequency sweep studies were carried out to determine *G′* and *G″* as a function of frequency (ω) at fixed temperature. *G′* is a measure of the deformation energy stored in the sample during the shear process, and it represents the elastic behavior of the hydrogel, therefore, any drop in *G′* represents the breakdown of the hydrogel structure. The *G″* value measures the deformation energy used up in the hydrogel during shear and lost afterwards from the hydrogel. The *G’’* value represents the viscous behavior of a hydrogel. Typically, outside the linear region, *G’’* starts to increase as the hydrogel becomes more fluid-like when the structure (*G′*) is broken down. In general, it is possible to state that if *G′* > *G″* the hydrogel has a solid-like behavior, while if *G′* < *G″* a liquid-like behavior is envisaged [36]. Using the values of *G′* and *G″*, the complex shear modulus (G*) can be obtained according to the following Equation:

(1) $$G*=\sqrt{{\left(G^{\prime }\right)}^{2}+{\left(G^{\prime \prime }\right)}^{2}}$$

The G* value can be considered as the overall resistance to deformation of a hydrogel, regardless of whether the deformation is recoverable (elastic) or non-recoverable (viscous). Complex modulus is a parameter to measure the rigidity of a material’s soft solid structure when exposed to stresses. Therefore, the G* value is a suitable indicator of visible features such as the flexibility or stiffness of a hydrogel.

**Figure 8:**
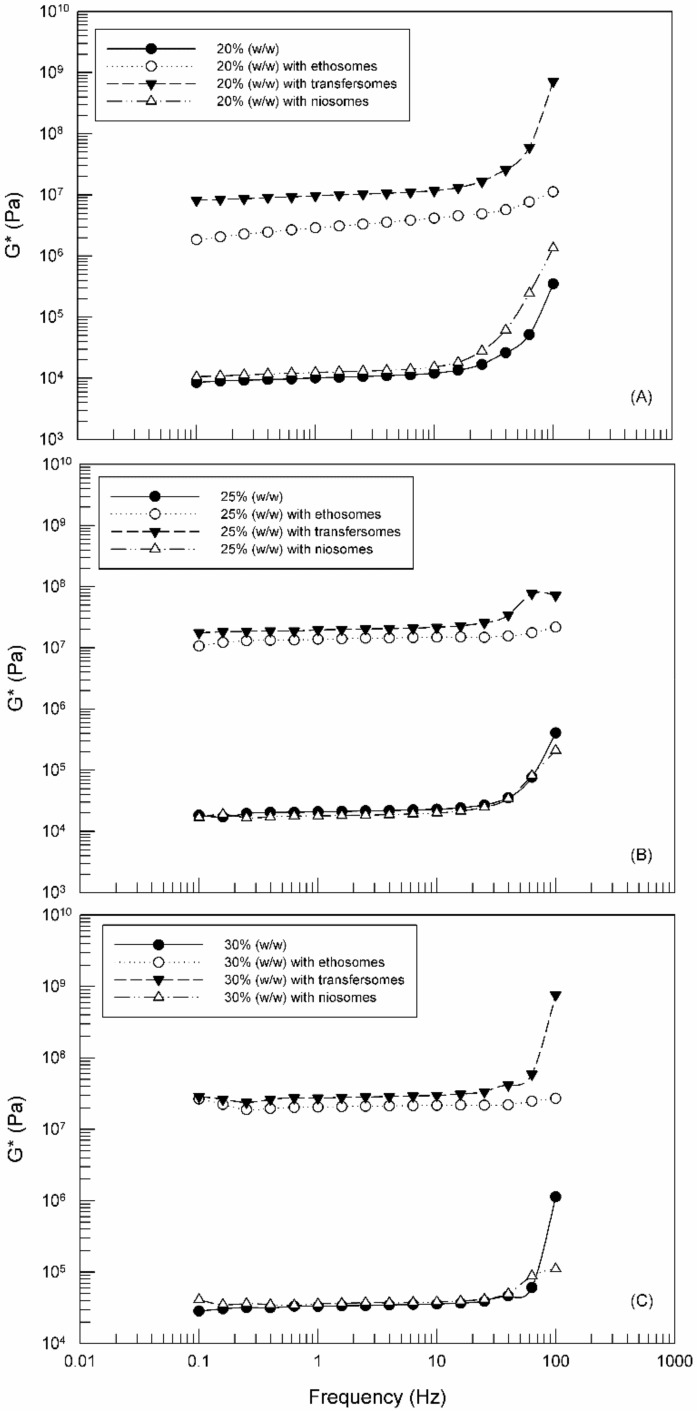
Complex shear modulus (G*) versus Frequency for 20% Poloxamer 407 (**A**), 25% Poloxamer 407 (**B**) and 30% Poloxamer 407 (**C**) with and without TDDSs. The illustrated results were representative of three independent experiments.

Figure 8 shows the effects of the composition and the presence of TDDSs on the resistance to deformation of Poloxamer 407 hydrogels. Comparing the three panels, the G* values increased with the increase of Poloxamer 407 concentration, similarly to that observed for viscosity.

The effects of TDDSs were different as a function of the nanocarriers. Niosomes did not affect the gel ability to withstand the applied stress. On the other hand, ethosomes and transfersomes were able to strengthen the gel network, as evidenced by G* values of poloxamer hydrogels containing ethosomes or transfersomes, which were greater than those observed in the case of unloaded hydrogel, independently on the poloxamer concentration (*p*-value < 0.001). This finding may be considered in disagreement with results reported in Figure 7, but probably the presence of ethosomes and transfersomes induced a decrease of viscosity making the gels more malleable and also more elastic and hence more resistant to break. These results are really promising, because these hydrogel types can suitably withstand the stress coming from the application on the skin.

### 2.5. Percutaneous Permeation Studios of Paclitaxel Loaded-Ethosomes, Transfersomes and Niosomes, Included in Poloxamer Gel

Paclitaxel was chosen as model lipophilic drug [38] to evaluate the effect of poloxamer gel on the ability of topical drug delivery systems to permeate through the skin and then to release their content. The entrapment of drug (0.666 mg/mL) into TDDSs led to an increase of mean size, while maintaining a good polydispersity index (Table 4). These results suggested that paclitaxel was incorporated in the lipid bilayer of nanosystems. About EE (%), ethosomes, transfersomes and niosomes have proven to be adept at containing high percentages of Paclitaxel, as show in Table 4. This result was foreseeable since these topical nanosystems prefer lipophilic drugs to hydrophilic ones.

After physicochemical characterization, paclitaxel-loaded topical nanosystems were introduced within poloxamer gel and the percutaneous permeation of paclitaxel through human skin was evaluated. In general, the efficacy of formulation for topical application depends on the ability of the loaded drug to permeate the skin and to accumulate in specific region of skin, for exerting its pharmacological action. Not all drugs are able to cross the skin layers, because a sufficient lipophilicity to partition in the stratum corneum is required and, at the same time, a sufficient hydrophilicity is necessary to allow the drug to partition into the viable epidermis [39,40].

**Figure 9:**
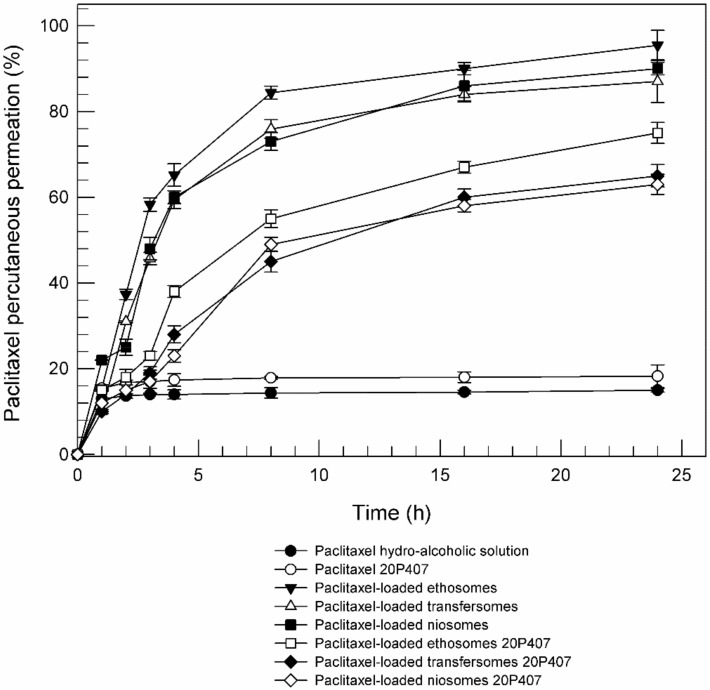
In vitro percutaneous permeation of paclitaxel from different formulations through SCE membranes, in comparison with a hydroalcoholic drug solution (as the control). Values represent the mean of three different experiments ± standard deviation.

The use of topical drug delivery systems can improve the percutaneous permeation of those drugs that do not have the suitable characteristics. According to previous studies, ethosomes, transfersomes and niosomes led to an enhanced percutaneous permeation effect of loaded paclitaxel in comparison with hydro-alcoholic solution of drug (Figure 9) [41,42]. The mechanisms responsible for improvement drug penetration across the skin by using topical drug delivery systems were widely described in the literature; in particular the ethanol included into ethosomes formulation seems to act as permeation enhancer on lipid structure of stratum corneum [9]; Cevc et al. suggested that sodium collate included into transfersomes act as an edge activator by decreasing the interfacial tension and hence increasing the deformability of the vesicle bilayers [43]; and finally, niosomes seem to be able to induce changes in the intercellular lipids reducing the stratum corneum barrier. However, all these effects on the skin structures are reversible and temporary [44].

Also the percutaneous permeation of paclitaxel-loaded TDDSs included into 20% (*w*/*w*) poloxamer gel was evaluated and reported in Figure 9. Independently of nanosystems, the permeation is slowed down in presence of poloxamer gel. The slowing down of the percutaneous permeation could be due to the reduced mobility of the nanosystems within the gel and therefore to a slower interaction of nanosystems’ components with the lipid structure of the skin. In any case the combination of poloxamer gel with topical drug delivery systems does not prevent a better partitioning of the drug, in comparison with the hydro-alcoholic solution of paclitaxel.

Moreover, Figure 9 reported that the poloxamer gel alone (Paclitaxel 20P407) is not able to improve the percutaneous permeation of paclitaxel. These findings suggest that TDDSs included in three-dimensional network of poloxamer gel could be used to achieve a long-time release of lipophilic drugs, such as paclitaxel.

## 3. Materials and Methods

### 3.1. Chemicals

Phospholipon 90G^®^ (PL-90G^®^) (93.0 ± 3.0% phosphatidylcholine) was obtained from Natterman Phospholipid GmbH (Cologne, Germany), and it was used without any purification. Absolute ethanol, chloroform, methanol, sodium cholate (SC), cholesterol (Chol), Tween 20 and Poloxamer P407 were purchased from Sigma Aldrich (Milan, Italy). Paclitaxel (HPLC purity 99%) was a gentle gift of Indena S.p.A. (Milan, Italy). All other materials and solvents used in this investigation were of analytical grade. Deionized double-distilled water was used throughout the investigation.

### 3.2. Methods

#### 3.2.1. Ethosomes Preparation

Three different batches of ethosomes were prepared according to the method previously reported by Paolino et al. [17]. Briefly, ethosomal suspensions were made up of 1% (*w*/*w*) PL-90G^®^ and 30% (*w*/*w*) absolute ethanol. PL-90G^®^ was dissolved with absolute ethanol and then hydrated with water, which was added slowly (200 μL/min) under a constant mixing with a magnetic anchor at 700 rpm (Midi MR1 Digital IkamagR; IKA-WERKE GmbH and Co., Staufen, Germany). Pyrex^®^ glass vials (hermetically sealed to avoid, as much as possible, the evaporation of ethanol) were used for ethosome preparation. The obtained ethosomes were then homogenized at 15,000 rpm for 3 min using an Ultra-Turrax T 25 equipped with an S25 N-8G homogenizing probe (IKA-WERKE) and then left at room temperature for 30 min under continuous stirring (Orbital Shaker KS 130 Control, IKA-WERKE).

#### 3.2.2. Transfersomes Preparation

Three different batches of transferosomes, also called ultradeformable liposomes, were prepared by dissolving PL 90G^®^ and SC with 1 mL of ethanol in a Pyrex^®^ glass vial. The organic solvent was removed by a Rotavapor^®^ (R210, Büchi–Italia, Milan, Italy) under a slow nitrogen flux, thus forming a thin lipid film on the inner wall of the vial, and then stored overnight at 30 °C in a Büchi T51 glass drying-oven under vacuum. The lipid film was then hydrated with 6 mL of a water/ethanol (93/7 *v*/*v*) solution and vortex-mixed at 700 rpm for 15 min at room temperature. Ultradeformable liposomes were left at 40 °C for 2 h and extruded as previously describe for liposomes [25].

#### 3.2.3. Niosomes Preparation

Three different batches of niosomes were prepared in part as described by Primavera et al. [45]. Briefly, vesicles were carried out using the thin layer evaporation method. The Tween 20 and CHOL (1:1 molar ratio) were dissolved in the organic mix of chloroform/methanol (3:1 molar ratio). The organic solvent was removed by evaporation through a R210 rotary evaporator (Büchi-Italia) under slow nitrogen flux, thus allowing the formation of a thin layer film on the inner wall of a round-bottomed flask. Any trace of residual solvent was eliminated by overnight storage at room temperature in a Büchi T51 glass drying oven connected to a vacuum pump. For the film hydration, a new method has been applied: Hepes buffer (10 mM, pH 7.4) has been added to the film and the mix has been vigorous mixed by vortexing at 11× *g* (Vortex, VELP Scientifica, Usmate (MB), Italy). Niosomes have been finely homogenized (25000 rpm) for 5 min using an Ultra-Turrax T25 equipped with an S25N-8G homogenizing probe (IKA-WERKE), subsequently samples were left for 5 min at room temperature. The procedure was repeated twice.

#### 3.2.4. Poloxamer 407-Based Hydrogel Preparation

Poloxamer 407-based hydrogels were prepared as described by Choi et al. [27]. Briefly, the exact amount of poloxamer (from 15% to 30% *w*/*w*) was dissolved in cold water maintaining a continuous stirring and a temperature of 4 °C. The liquid hydrogel was left at 4 °C overnight until a clear solution was obtained. To prepare gel formulation with nanosystems, ethosomes, transfersomes and niosomes were concentrated by using ultracentrifugation. In detail, nanosystems dispersions were poured into polycarbonate tubes and then centrifuged at 90,000× *g* for 1 h at 4 °C using an Avanti 30 centrifuge (Beckman, Fullerton, CA, USA) equipped with a fixed angle rotor Beckman mod. F1202. The supernatant and the pellet were divided. The obtained semisolid mass of nanosystems was add to the gel formulation (2% *w*/*w*) for subsequent studies.

#### 3.2.5. Physicochemical Characterization of Vesicles Formulations

Mean size, size distribution and z-potential were evaluated using a Zetasizer Nano ZS (Malvern Instruments Ltd., Worchestershire, UK), which is a dynamic light-scattering spectrophotometer [46]. A third-order cumulant fitting was applied as a correlation function. The instrument used a 4.5 mW laser diode operating at 670 nm for size analysis and back-scattered photons were detected at 173°. The medium refractive index (1.330), medium viscosity (1.0 mPa × s), and dielectric constant (80.4) were set before the experiments. Polycarbonate cuvettes were used for the analysis.

The stability of the carriers was determined by using a Turbiscan Lab^®^ Expert [25], equipped with a Turbiscan Lab Cooler. Briefly, the samples were placed into a cylindrical glass tube and measurements were carried out for 1 h. The photon which was transmitted (T) and backscattered (BS) was recorded. Analysis was carried out at the temperature of 24 ± 1 °C. The analyses were performed using the TurbiSoft software (Formulaction, L’Union, France) and the obtained data were used to evaluate the kinetic stability of the formulations.

#### 3.2.6. Determination of Gelation Temperature of Poloxamer 407-Hydrogles

In a beaker, 10 gr of poloxamer solution were placed on the thermostat stirring plate and a digital thermosensor (Ika Labortechnik, RET digi-visc) connected to a thermistor was immersed in the poloxamer solution. Poloxamer solution was heated at a constant rate with constant stirring. When the magnetic bar stopped moving due to gelation, the temperature displayed on the thermistor was recorded as a gelation temperature [27].

#### 3.2.7. Diffusing Wave Spectroscopy (DWS)

The measurements of the microrheology of Poloxamer 407-based hydrogels, with and without carriers, were carried out by using the diffusing wave spectroscopy (DWS) (Rheolaser Master™, Formulaction), thus measuring the particles’ Brownian motion which depends on the viscoelastic feature of our sample. Sample (20 mL) microrheology was measured by adding latex beads (1 μm, 0.1 wt. %).

Micro-rheology measurements were performed for 1 h and were carried out on each hydrogel sample prepared with different percentage of poloxamer 407. In detail, samples were placed into glass vials thermostated to a temperature of 4 °C, vials were placed in the instrument sample-holder and backscattered light intensity was detected at 25 °C. In this way, it was possible to assess the gelation rate at 25 °C.

Thanks to the microrheological analysis carried out by the Rheolaser Master™, mean square displacement (MSD) was obtained for all samples. MSD was used to determine the sample particle movement as a function of time. Namely, the MSD value is the average of a number of scattering trajectory where the unit is run.

The microrheological parameters, namely mean square displacement curves, elasticity index (EI) and solid liquid balance (SLB), were collected and processed by the RheoSoft Master 1.4.0.0. [28]. EI value is the sample elasticity strength and corresponds to the MSD slope at a short decorrelation time. SLB is the ratio between the solid-like and the liquid-like behavior of the sample. Namely, a 0.5 < SLB < 1 means that the liquid behavior dominates, while a 0 < SLB < 0.5 means that the solid behavior dominates thus defining it as gel behavior.

#### 3.2.8. Hydrogel Dynamic Rheological Characterization

Kinexus Pro+ rotational rheometer (Malvern Instruments Ltd. Worchestershire, UK), equipped with cone-plate geometries (40 mm diameter; 2° angle), was used for the sample rheological characterization. All runs were carried out at 25.00 ± 0.01 °C. A fixed gap between the geometries was pre-set to 1 mm and the excess sample was removed. The rSpace software processed rheological data. Compressed air flow (2 bar), pre-filtered through fine and superfine Clearpoint filter (Beko, Atlanta, GA, USA), allowed reaching the pressure able to perform the analysis. The samples were carefully and gently loaded onto the measuring plate of the rheometer and the measuring geometry was lowered at very slow speed, in order to prevent the alteration of sample structure [47]. Hydrogel samples were rested 15 min before each analysis. Runs were carried out to evaluate the influence of both the different percentage of Poloxamer 407 used during hydrogels preparation and the presence of colloidal carriers on hydrogel rheological features.

The rheological characterization involved the study of viscosity curves and oscillation test. The viscosity curves were obtained by means of a shear rate ramp starting from 0.1 s^−1^ and ending to 100 s^−1^. The values of dynamic viscosity at 0.1, 1, 10 and 100 s^−1^ were used to characterize hydrogel samples. While, oscillatory test with a frequency sweep ranging from 0.01 Hz to 100 Hz was carried out at controlled stress (1 Pa). The dynamic rheological properties of hydrogels were evaluated in terms of storage modulus *G′* (elastic component), loss modulus *G″* (viscous component) and complex shear modulus (G*), thus evaluating the significant differences between hydrogel samples prepared using different percentage of poloxamer 407 either containing or not colloidal carriers.

#### 3.2.9. Preparation of Paclitaxel-Loaded TDDSs and Percutaneous Permeation Studies

To evaluate the influence of poloxamer gel on percutaneous permeation of a drug-loaded nanosystems, ethosomes, transfersomes and niosomes were prepared solubilizing Paclitaxel (0.666 mg/mL), in the organic phases. Paclitaxel was used as model drug, based on previous experiences [48,49]. After physicochemical characterization (see Section 3.2.5.), the nanosystems were centrifuged at 90,000 rpm for 1 h using a Beckman Optima™ Ultracentrifuge (Beckman, Fullerton, CA). The obtained pellets were dissolved in cold methanol and the amount of drug contained in the pellet was analyzed by HPLC [32]. The entrapment efficacy (EE) of ethosomes, transfersomes and niosomes was determined by using the following Equation:

(2) $$EE=\left(\frac{De}{Dt}\right)\times 100$$

where *Dt* is the amount of drug added the preparation of paclitaxel-loaded TDDSs, and *De* is the amount of drug contained in the pellet.

To evaluate the in vitro percutaneous permeation of paclitaxel entrapped in ethosomes, transfersomes and niosomes, and included in the poloxamer gel, dynamic skin permeation systems and human stratum corneum and viable epidermis (SCE) membranes were used [50]. The in vitro experiments were performed in accordance with the Declaration of Helsinki, and the protocol was approved by the Research Ethics Committee of the University Magna Græcia of Catanzaro (Approval number: 390/2019). SCE membranes were obtained from fresh abdominal human skin resulting from plastic reduction surgery of healthy adults (mean age 40 ± 5 years).

Percutaneous permeation experiments were carried out in non-occlusive conditions and SCE membranes have been positioned between donor and receptor chamber of skin permeation systems, also called dynamic Franz cells. Moreover, the donor was filled with tested samples (200 µL), and the receptor, characterized by a nominal receiving volume of 4.75 mL, was filled with an ethanol/water mixture (20:80 *v*/*v*). Experimental conditions were maintained at 37 ± 0.5 °C and continuously stirred at 100 rpm using a magnetic stirrer. During 24 h of experiments, 500 µL of the receptor phase was collected for HPLC analysis at prefixed time intervals. The amount of withdrawn receptor solution was replaced with 500 µL of fresh solution. Experiments were carried out in triplicate and the results were the average of three different experiments ± standard deviations.

#### 3.2.10. Statistical Analysis

One-way ANOVA was used for statistical analysis of the various experiments. A posteriori Bonferroni t-test was carried out to check the ANOVA test. A *p*-value < 0.05 was considered statistically significant.

## 4. Conclusions

This investigation demonstrated that the presence of TDDSs in the three-dimensional network of Poloxamer 407 hydrogels was not detrimental for them in terms of rheological features, such as spread-ability. Our findings support the idea to use a combination of TDDSs and Poloxamer 407 hydrogels to boost the topical delivery of drugs. In fact, the topical application of vesicular nanocarriers embedded in poloxamer hydrogels for percutaneous drug delivery may lead to different advantages: (i) the achievement of a synergistic effect between nanocarriers and hydrogel matrix thus favoring the passage of active ingredients, which are normally not able to cross the stratum corneum; (ii) allow a more controlled release of the drug and (iii) the increase of the persistence time of nanocarriers on the skin.

Moreover, the so far obtained data were very encouraging and demonstrated that the Rheolaser Master™ and Kinexus Rotational Rheometer are valid, not time-consuming and complementary tools for studying the rheological properties of topical gels and particularly of those formulations characterized by the presence of vesicular systems into a three-dimensional network. Furthermore, thus predicting their potential topical applications and hence allowing a fine design of topical dosage forms to fulfill the application requirements for both suitable therapeutic and cosmetic responses.

## Figures and Tables

**Table 1:** Physico-chemical parameters of drug delivery systems obtained by use Zetasizer Nano ZS. Values are reported as the average of three independent experiments ± standard deviation.

Sample	Mean Size (nm)	Polydispersity Index	Zeta-Potential (mV)
Ethosomes	200.00 ± 4.43	0.16 ± 0.01	−15.20 ± 0.38
Transferosomes	187.90 ± 1.87	0.24 ± 0.01	−29.50 ± 0.59
Niosomes	123.50 ± 1.01	0.22 ± 0.01	−26.00 ± 0.35

**Table 2:** Shear rate values correspondent to yield points for 20%, 25% and 30% poloxamer samples. Values are reported as the average of three independent experiments ± standard deviation.

Sample	Shear Rate (s^−1^)
20% Poloxamer 407	0.1585 ± 0.0015
25% Poloxamer 407	0.2512 ± 0.0003
30% Poloxamer 407	0.6310 ± 0.0032

**Table 3:** Shear rate-dependent viscosity (Pa·s) for Poloxamer 407 gels at 25 °C and at different shear rates. Values are reported as the average of three independent experiments ± standard deviation.

Sample	Shear Viscosity (Pa·s) at Different Shear Rate			
	0.1 s^−1^	1 s^−1^	10 s^−1^	100 s^−1^
20% Poloxamer 407	1618000.0 ± 230.1	173.5 ± 10.6	19.7 ± 2.5	2.6 ± 0.6
25% Poloxamer 407	3577000.0 ± 307.6	434.3 ± 7.9	52.8 ± 1.6	6.3 ± 0.9
30% Poloxamer 407	5708000.0 ± 98.7	610.1 ± 24.0	75.9 ± 2.0	9.7 ± 1.0

**Table 4:** Physico-chemical parameters of paclitaxel-loaded drug delivery systems obtained by use Zetasizer Nano ZS. Values are reported as the average of three independent experiments ± standard deviation.

Sample	Mean Size (nm)	Polydispersity Index	EE (%)
Ethosomes	309.00 ± 2.51	0.19 ± 0.01	65.54 ± 1.47
Transferosomes	265.07 ± 19.00	0.56 ± 0.01	57.27 ± 1.03
Niosomes	218.50 ± 7.53	0.32 ± 0.01	42.5 ± 0.35

## Supplementary Materials

The following are available online: Figure S1: Mean square displacement MSD for 25% Poloxamer 407 gel without carriers (A), or with ethosomes (B), transfersomes (C) and niosomes (D) as a function of correlation. The illustrated results are one of the parallel experiments. Figure S2: Elasticity Index (EI) and Solid Liquid Balance profile versus time for 25% (w/w) Poloxamer 470 with and without TDDSs. Figure S3: Mean square displacement MSD for 30% Poloxamer 407 gel without carriers (A), or with ethosomes (B), transfersomes (C) and niosomes (D) as a function of correlation. The illustrated results are one of the parallel experiments. Figure S4: Elasticity Index (EI) and Solid Liquid Balance profile versus time for 30% (w/w) Poloxamer 470 with and without TDDSs. Table S1 Gelation temperature of Poloxamer 407 solutions. Each value represents the mean ± S.D. of three experiments.
