# Supplementary material for: The Rheolaser Master™ and Kinexus Rotational Rheometer® to Evaluate the Influence of Topical Drug Delivery Systems on Rheological Features of Topical Poloxamer Gel
Source: Molecules. 2020 Apr 23;25(8):1979. doi: 10.3390/molecules25081979 (PMC7221562; doi:10.3390/molecules25081979)
Supplement: Supplementary file 1 [file molecules-25-01979-s001.pdf]

# The Rheolaser Master™ and Kinexus Rotational Rheometer® to Evaluate the Influence of Topical Drug Delivery Systems on Rheological Features of Topical Poloxamer Gel

**Maria Chiara Cristiano [1], Francesca Froiio [1], Antonia Mancuso [2], Federica De Gaetano [3], Cinzia Anna Ventura [3], Massimo Fresta [2] and Donatella Paolino [1,*]**

[1] Department of Experimental and Clinical Medicine, School of Pharmacy and Nutraceuticals, University "Magna Græcia" of Catanzaro, Campus Universitario "S. Venuta"-Building of BioSciences, Viale S. Venuta, I-88100 Germaneto-Catanzaro, Italy; mchiara.cristiano@unicz.it (M.C.C.); f.froiio@unicz.it (F.F.);

[2] Department of Health Sciences, School of Pharmacy and Nutraceuticals, University "Magna Græcia" of Catanzaro, Campus Universitario "S. Venuta"- Building of BioSciences, Viale S. Venuta, I-88100 Germaneto-Catanzaro, Italy; antonia.mancuso@unicz.it (A.M.); fresta@unicz.it (M.F.)

[3] Department of Chemical, Biological, Pharmaceutical and Environmental Sciences, University of Messina, Viale Ferdinando Stagno D'Alcontres 31, Messina I-98166, Italy; fedegaetano@unime.it (F.D.G.); caventura@unime.it (C.A.V.)

* Correspondence: paolino@unicz.it; Tel.: +39+0961+369+4118; Fax: +39+0961+369+4237

Table S1 Gelation temperature of Poloxamer 407 solutions. Each value represents the mean ± S.D. of three experiments.

| Poloxamer 407 concentration (%, w/w) | Gelation Temperature (°C) |
|---|---|
| 15 | 37 ± 0.5 |
| 17 | 36 ± 0.5 |
| 20 | 21 ± 0.2 |
| 25 | < 20 |
| 30 | < 20 |

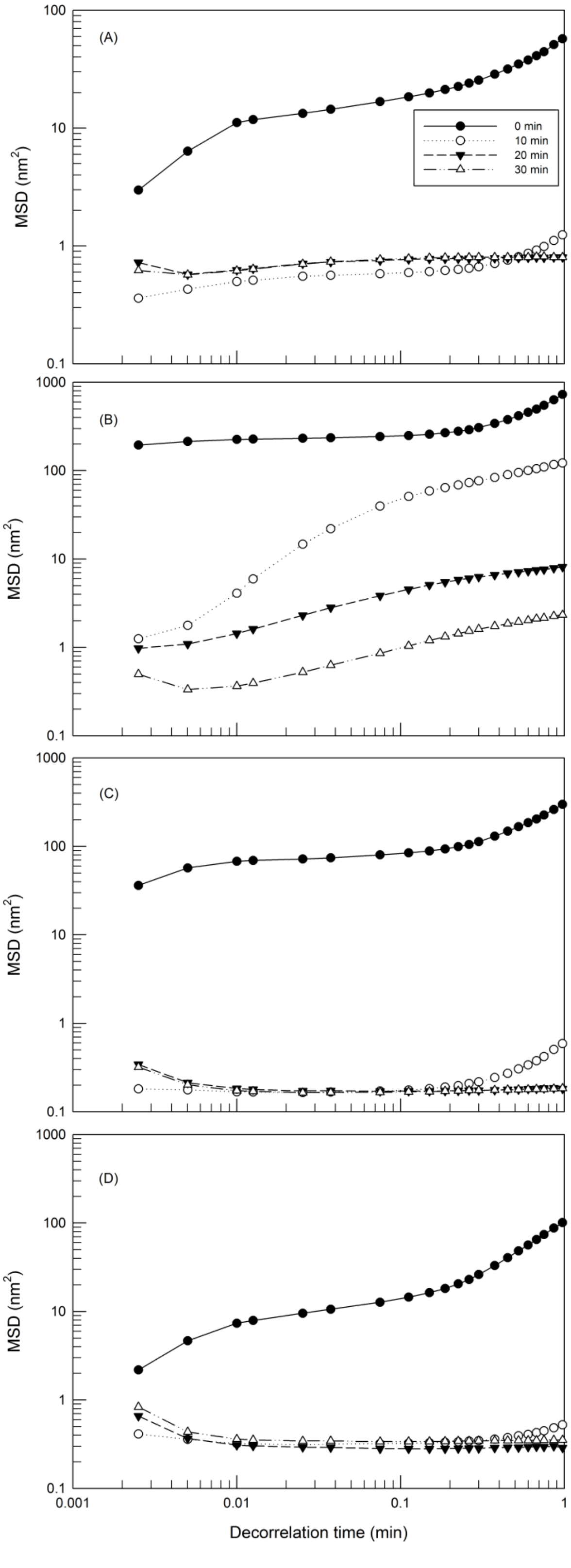


Figure S1: Mean square displacement MSD for 25% Poloxamer 407 gel without carriers (A), or with ethosomes (B), transfersomes (C) and niosomes (D) as a function of decorrelation time. The illustrated results are one of the parallel experiments.

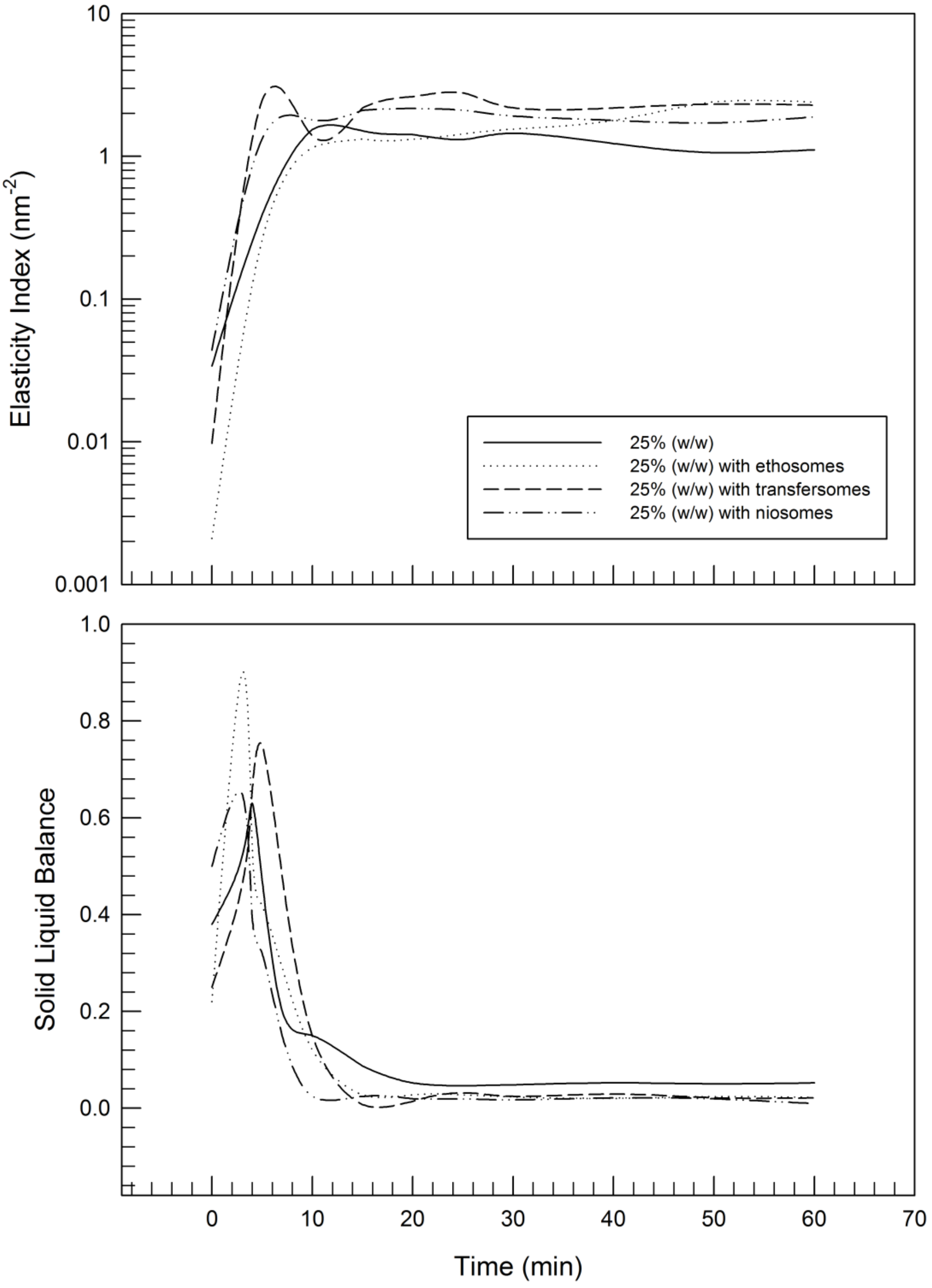


Figure S2: Elasticity Index (EI) and Solid Liquid Balance profile versus time for 25% (w/w) Poloxamer 470 with and without TDDSs.

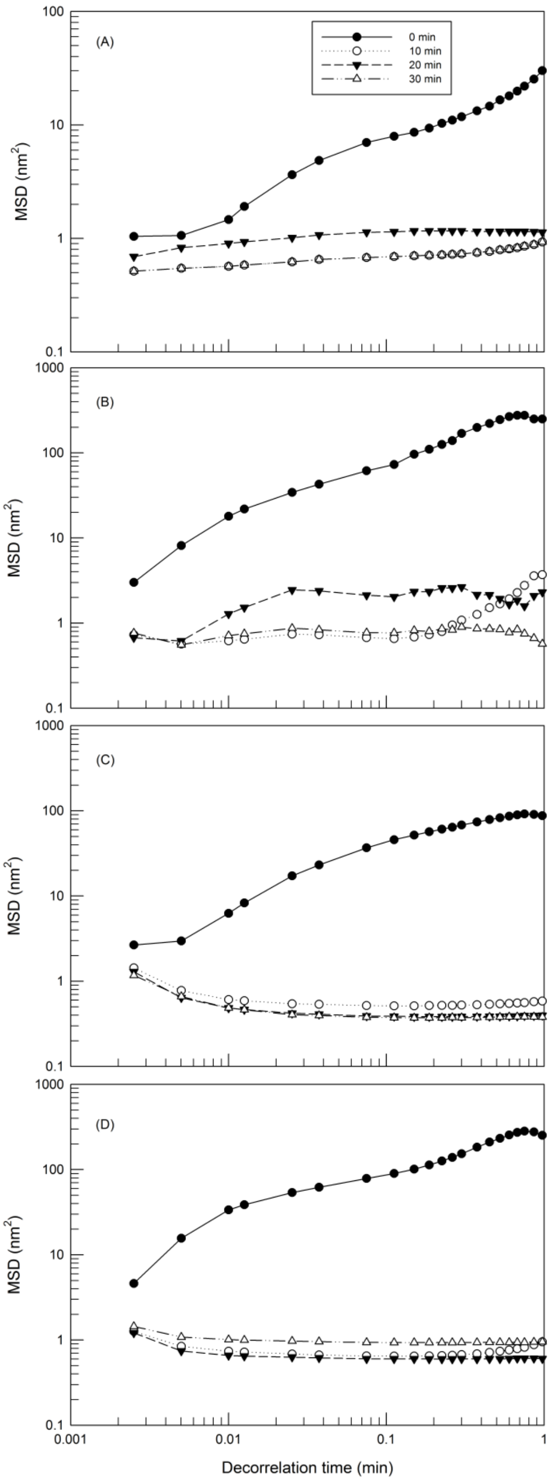


Figure S3: Mean square displacement MSD for 30% Poloxamer 407 gel without carriers (A), or with ethosomes (B), transfersomes (C) and niosomes (D) as a function of decorrelation time. The illustrated results are one of the parallel experiments.

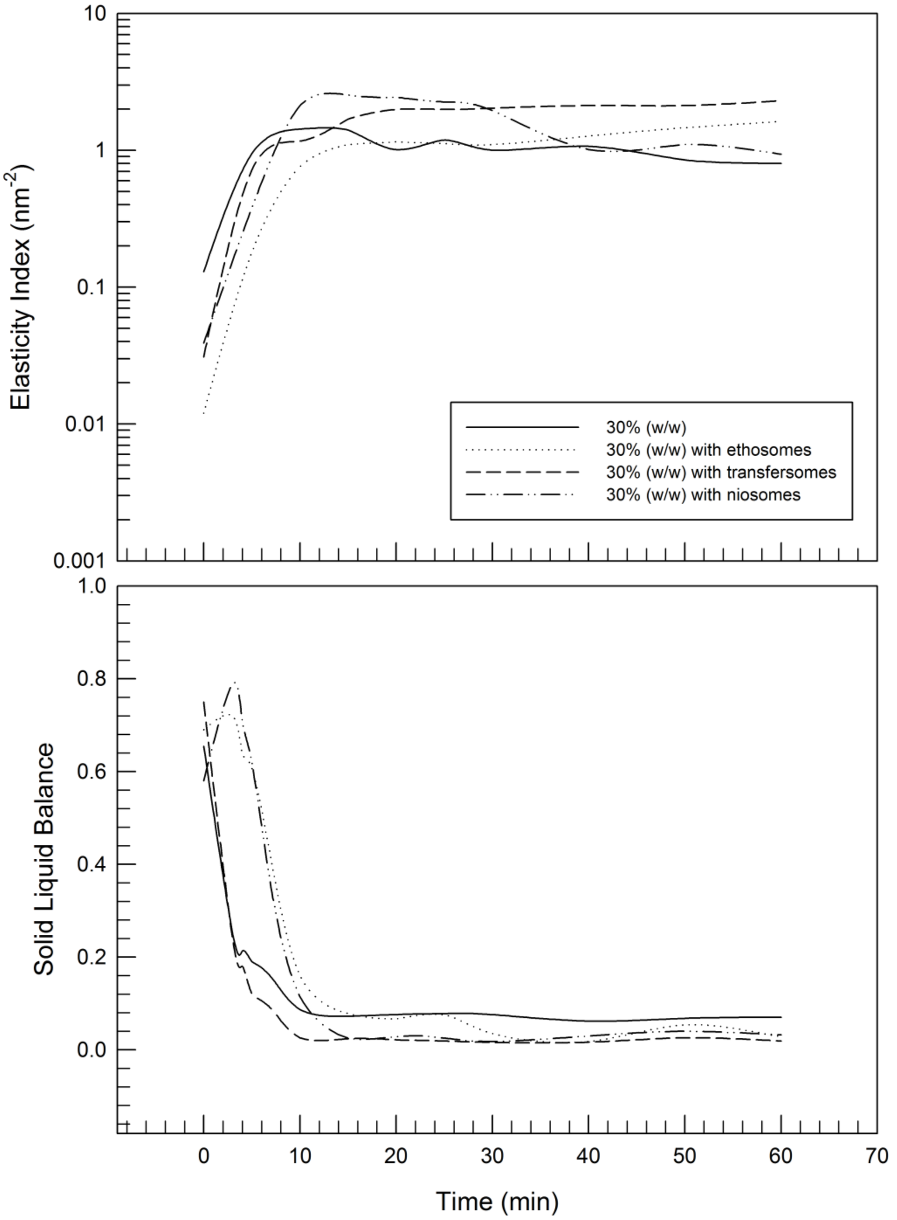


Figure S4: Elasticity Index (EI) and Solid Liquid Balance profile versus time for 30% (w/w) Poloxamer 470 with and without TDDSs.
